# The Physical Activity Levels and Sedentary Behaviors of Latino Children in London (Ontario, Canada)

**DOI:** 10.3390/ijerph120505528

**Published:** 2015-05-22

**Authors:** Gillian Mandich, Shauna Burke, Anca Gaston, Patricia Tucker

**Affiliations:** Faculty of Health Sciences, University of Western Ontario, London ON N6A 5B9 Canada

**Keywords:** physical activity, sedentary behavior, children, Latino

## Abstract

*Objective:* To assess the physical activity and sedentary behaviors of a sample of Latino children in London, Ontario, Canada. *Methods:* Seventy-four Latino children (54.1% male; mean age = 11.4) completed self-report questionnaires related to physical activity and sedentary behaviors. A subset of children (*n* = 64) wore Actical (Mini Mitter, Respironics) accelerometers for a maximum of four days. *Results:* Latino children self-reported moderate levels of physical activity (*i.e.*, mean score of 2.8 on 5-point scale). Accelerometer data revealed that children spent an average of 50.0 min in moderate-to-vigorous physical activity (MVPA; 59.2 min on weekdays and 50.6 min on weekend days) and were sedentary for an average of 8.4 h (508.0 min) per day (533.5 min on weekdays and 497.7 min on weekend days). Children reported spending an average of 3.8 h (228 min) daily in front of screens—1.7 h (102 min) watching television, 1.2 h (72 min) on the computer, and 0.9 h (54 min) playing video games. *Conclusions:* This feasibility project provided a preliminary account of objectively measured daily physical activity and sedentary time among a sample of Latino children in Canada, as well as insight into the challenge of measuring these behaviors. Sedentary behavior reduction techniques should be explored and implemented in this young population, along with strategies to promote adherence to accelerometer protocols.

## 1. Introduction

Physical activity is associated with a wide range of health benefits for children [1]. With regard to physical health, increased levels of physical activity are associated with improved body composition and increased cardiorespiratory fitness [2,3]. Insofar as psychosocial health is concerned, research has shown that increased levels of physical activity among children may help to reduce anxiety and depression, as well as improve self-esteem [4].

The most recent Canadian Physical Activity Guidelines for children were released in 2011 [5], suggesting that children and youth should accumulate at least 60 min of moderate to vigorous intensity physical activity (MVPA) per day. When using objective measures of physical activity, it is evident that most children in North America fail to meet this recommendation. In Canada, accelerometer data (using the Actical device, Mini Meter, Bend, OR, USA) from the 2007–2009 Canadian Health Measures Survey (CHMS) showed that only 7.0% of Canadian children and youth aged 5 to 17 accumulated 60 min of MVPA on a daily basis [6]. In the United States, accelerometer data (using the ActiGraph device [7]) from the 2003–2004 National Health and Nutrition Examination Survey (NHANES) revealed that less than half (*i.e.*, 42.0%) of children aged 6 to 11 acquired at least 60 min of MVPA on most days [8].

While the physical activity levels of children appear to be decreasing across the globe [9], mounting evidence suggests that sedentary behaviors (including screen time) are associated with their own adverse health outcomes [10]. As such, in 2011, the Canadian Society for Exercise Physiology published guidelines [5] that recommended that children and youth between the ages of 5 and 17 should limit recreational screen time to less than two hours per day, and minimize motorized transportation, time spent sitting, and time spent indoors.

When examining Latino children specifically, research conducted in the United States has documented trends of low levels of physical activity that persist into adulthood [11]. Recently, Fakhouri and colleagues examined the proxy-reported physical activity and screen-viewing behaviors of children aged 6 to 11 from the 2009–2010 NHANES [12]. Interestingly, these researchers concluded that Hispanic children: (a) were less likely to meet current physical activity guidelines than non-Hispanic white children; and (b) spent less time engaged in screen-viewing behaviors than non-Hispanic white children [12].

Additionally, in a sample of 483 Latino children between the ages of 9 and 12 years, only 23% of the children met the MVPA recommendation [13], and findings from Trost and colleagues in 2013 found that Latino children had lower levels of MVPA when compared with other racial/ethnic groups as only 19% of Latino children met the recommendation of daily MVPA [14].

With regard to sedentary behaviors specifically, and in contrast to the results presented by Fakhouri and colleagues, previous research has shown that Latino children in the United States spend *more* time engaged in screen-related sedentary behaviors than Caucasian children [12]. For example, in 2009, Sisson *et al.* [15] examined the self-reported sedentary behaviors of 8,708 children from the United States NHANES. Results revealed that a greater proportion of Latino children spent more than two hours per day in front of screens compared to their European American counterparts [15].

Given that Latinos comprise one of the largest non-European ethnic groups in Canada [16], it is important to examine the health-related behaviors of Latino children. Although there is ample evidence documenting low rates of physical activity among Latino children in the United States [11], this topic has not been addressed in Canada. Additionally, the literature related to sedentary time and screen-viewing behaviors among Latino children appears to be mixed [17]. Thus, the purpose of the present study was to examine the physical activity levels and sedentary time of a sample of Latino children (aged 10–14 years) in London, Ontario, Canada. In addition to using subjective (*i.e.*, self-report) assessments of physical activity and sedentary time, objective (*i.e.*, accelerometer) measures were used in an attempt to address the measurement limitations associated with using either approach alone. This study represents important preliminary work and can be defined as a feasibility study given that we also aimed to test the assessment procedures for their potential use in a future full-scale study with a larger sample [18].

## 2. Methods

### 2.1. Participants

All subjects gave their informed consent for inclusion before they participated in the study. The study was conducted in accordance with the Declaration of Helsinki, and the protocol was approved by the Ethics Committee of the University of Western Ontario (REB #17676.). The present study employed a cross-sectional research design to measure the physical activity levels and sedentary time of Latino children in London, Ontario, Canada. Inclusion criteria included being between the ages of 10 and 14 and physically able to participate in physical activity. Additionally, the child and his or her guardian had to: (a) self-identify as Latino (a term used to refer to individuals whose origins can be traced to countries that share the Spanish language as a common identifier such as the Caribbean, Mexico, Central America, and South America) [19]; (b) be willing to participate; and (c) be able to complete and comprehend written questionnaires (available in English or Spanish).

### 2.2. Procedures and Measures

All forms, questionnaires, and procedures were approved by the University of Western Ontario Research Ethics Board. Recruitment and data collection took place during the months of July and August 2011. Participants were recruited by a member of the research team with the assistance of a local non-profit organization that works with Canadians of Latino descent. Recruitment posters were placed at local Latino establishments and businesses, and participants were also recruited at cultural festivals and via snowball sampling. The snowball sampling technique has been used traditionally as a means of recruiting difficult-to-reach communities in programs or research studies [20]; in the current study, the caregivers of participants were asked if they knew any other families that might be interested in participating. If the family had more than one child, all children between the ages of 10 to 14 years were invited to participate.

Once the child and his or her family agreed to participate in the study, informed consent and assent were obtained from caregivers and participants, respectively. One caregiver per family was then asked to complete a brief sociodemographic questionnaire. Next, children filled out a modified version of the Physical Activity Questionnaire for Children (PAQ-C) [21], a self-report questionnaire that asked children to recall activities and behaviors from the previous seven days. Children also completed a modified version of the Child Sedentary Behaviour Questionnaire (CSBQ) [22,23], a tool that measured screen viewing and sedentary time in addition to contextual information pertaining to the types of sedentary behaviors in which children engaged. Because both the PAQ-C and CSBQ were designed to be administered during the school year, some questions were modified to reflect the fact that data collection was completed during the summer months.

Following completion of the questionnaires, children’s anthropometric data (height, weight, and waist circumference) were collected by a trained researcher. Body fat percentage was also recorded using a TANITA Body Composition Analyzer (TBF-410, Tokyo, Japan). All participants were asked to wear a Mini Mitter Actical accelerometer for four consecutive days (Thursday, Friday, Saturday, and Sunday) in order to objectively measure physical activity levels. The Actical is an “omnidirectional” tri-axial accelerometer that is typically worn on the hip and senses motion in all directions; it has been shown to be a valid and reliable predictor of physical activity and energy expenditure in youth [24].

### 2.3. Data Analysis

To calculate subjective physical activity scores, data from the modified PAQ-C were scored to obtain an activity score between 1 and 5 for each item [25]. To calculate a final modified PAQ-C activity summary score, a mean value from each of the questions on the modified PAQ-C was calculated. Children’s self-reported daily screen time was calculated by adding the total amount of time children reported engaging in different screen-related activities per day on the modified CSBQ.

The main variables of interest with regard to objectively measured activity (measured via accelerometers) were moderate to vigorous physical activity (MVPA) and sedentary time. Raw accelerometer data were recorded in 15-second epochs and analyzed using custom software (KineSoft, Version 3.3.62, Saskatchewan, Canada, a custom software program) to produce a series of standardized outcome variables [26]. Similar to protocol used by Esliger, Copeland, Barnes, and Tremblay, a “day” of accelerometer data was defined as valid if it included 10 or more hours of monitor wear time [6,26]. Consistent with procedures used by Vanderloo and colleagues [27] and Troiano and colleagues [8], participants with *one or more* valid days of accelerometer data were included in the analyses. Previously validated cut-points for children [6,28] were used to classify the data as sedentary (less than 0.01 kcal/kg/min), light (0.01 to less than 0.04 kcal/kg/min), moderate (0.04 to less than 0.10 kcal/kg/min), or vigorous (0.10 kcal/kg/min or more); MVPA was defined as any activity exceeding the moderate intensity threshold.

Insofar as the modified CSBQ was concerned, average values for both weekdays and weekend days as well as for boys and girls were calculated, and differences between these variables were examined using independent samples and paired *t*-tests. Differences in self-reported physical activity levels between boys and girls were examined using independent samples *t*-tests. All analyses for the Actical data were conducted using custom software (KineSoft, Version 3.3.62) and SPSS (version 17.0, SPSS Inc., Chicago, IL, USA). Actical data were examined based on sex as well as weekday and weekend days, and differences between these values were examined using independent samples and paired *t*-tests.

## 3. Results

A total of 74 Latino children completed the self-report questionnaires and anthropometric assessment, and a subset of 64 participants wore accelerometers (60 of which had at least one “valid day” of accelerometer data) and were subsequently included in analyses. Table 1 provides a description of participant characteristics. More than half of participants (54.1%; *n* = 40) were male and the mean age was 11.4 years (*SD* = 1.3).

**Table 1:** Participant characteristics.

Participant Characteristic	*N* (%)	Mean	*SD*	Minimum	Maximum
Age (years)	74	11.4	1.3	10.0	14.0
Sex					
Male	40 (54.1%)				
Female	34 (46.0%)				

Birthplace of child					
Canada	8 (10.8%)				
Columbia	53 (71.6%)				
Mexico	1 (1.4%)				
El Salvador	6 (8.1%)				
USA	4 (5.4%)				
Peru	1 (1.4%)				
BMI-*z* score **^a^**	74	0.6	1.2	-2.0	2.9
Waist circumference (cm)	74	74.5	15.3	25.9	112.5
Body fat (%) *	73	20.7	11.0	11.7	49.9
Fat free mass (%) *	73	77.1	14.5	15.4	98.3

Notes: BMI-*z* = Standardized Body Mass Index. **^a^** BMI-*z* is interpreted such that a score of zero is an average score and each score above or below zero is one standard deviation above or below normal. *Assessed using a TANITA Body Composition Analyzer (TBF-410, Japan).

### 3.1. Physical Activity Levels

#### 3.1.1. Subjective Measurement

Thirty-nine boys and 34 girls completed the modified PAQ-C. Overall, participants reported a mean activity score of 2.8 (*SD* = 0.6; min = 1.0, max = 4.7), out of a maximum score of 5, for the past seven days. With regard to sex, boys (activity score = 2.9, *SD* = 0.6) self-reported as significantly more active than girls (activity score = 2.6, *SD* = 0.6; *t*(72) = 2.1, *p =* 0.04). The scale reliability was acceptable for both females (Cronbach’s alpha (α) = 0.83) and males (α = 0.80).

#### 3.1.2. Objective Measurement

Table 2 contains data pertaining to objectively measured physical activity. Children (*n* = 64) wore the accelerometers for an average of 12.1 hours (*SD* = 1.7) per valid day. As noted above, 60 Latino children were included in the analysis because they had at least one valid day of wear time [8,12]. The number of children with a valid day of Actical wear time varied over the course of the four-day data collection period: 42 children on day 1 (Thursday), 40 on day 2 (Friday), 43 on day 3 (Saturday), and 28 on day 4 (Sunday). Fifteen children had a total of four days of valid accelerometer wear time, 15 had three valid days, 14 had two valid days, and 16 children had only one day of valid wear time.

#### 3.1.3. Moderate-to-Vigorous Physical Activity (MVPA)

Overall, children spent an average of 50.0 min (*SD* = 34.2) per valid day engaged in MVPA. On average, boys spent more time per valid day (53.9 min, *SD* = 34.2) engaged in MVPA, compared to their female counterparts (48.2 min, *SD* = 35.7); this trend was borderline significant (*t*(58) = 0.6, *p* = 0.054). Almost all MVPA (90.4%; 47.8 min, *SD* = 33.3) was accumulated at a moderate intensity.

In terms of the amount of MVPA children engaged in on a daily basis, only 15 children had four days of valid accelerometer wear time; of these, only one (male) engaged in 60 min or more on all four days. Approximately half of boys (51.7%, *n* = 16) and girls (44.8%, *n* = 13) achieved 60 min of MVPA on at least one of the four valid days.

### 3.2. Sedentary Behaviors

#### 3.2.1. Subjective Measurement

A total of 73 children completed the modified CSBQ [23]. The mean amount of time children reported engaging in screen-related sedentary behaviors was 3.8 h (*SD =* 2.0) per day. Of those, 1.8 h (*SD =* 1.4) were reportedly spent watching television, 1.2 h (*SD =* 1.2) were spent on the computer, and 0.9 h (*SD =* 1.0) were spent playing video games. There was no significant difference (t(59) = 1.65, *p* = 0.10) in the amount of time children spent engaged in sedentary behaviours on weekdays (8.9 h, *SD* = 1.6) *versus* weekend days (8.4 h, *SD* = 1.3).

Overall, boys reported spending more time (3.9 h, *SD* = 1.9) engaged in screen-related sedentary behaviors than girls (3.7 h, *SD* = 2.1), although this difference was not statistically significant (*t*(71) = 0.4, *p* = 0.8). With regard to self-reported total daily screen time, 66.7% of boys (*n* = 26) and 61.8% of girls (*n* = 21) accumulated more than two hours of screen time per day, and 48.7% (*n* = 19) of boys and 34.2% (*n* = 12) of girls accumulated more than four hours of screen time per day. The scale reliability was acceptable for both females (Cronbach’s alpha (α) = 0.73) and males (α = 0.74).

#### 3.2.2. Objective Measurement

Accelerometer data revealed that children were sedentary for an average of 8.1 h (*SD* = 1.4) per day (see Table 2). Participants spent 8.9 h (*SD* = 1.6) engaged in sedentary behaviours on weekdays and 8.4 h (*SD* = 1.3) on weekends.

Given participants’ monitor wear time, 70.8% of children’s time per observed day was spent being sedentary. Girls were slightly more sedentary than boys (8.8 h per day, *SD* = 1.4, *vs.* 8.4 h per day, *SD* = 1.5, respectively), although the difference was not statistically significant (*t*(58) = 0.9, *p* = 0.4).

**Table 2:** Objectively assessed Physical Activity (PA) and sedentary time of Latino children on valid days, measured via actical accelerometers.

Amount and intensity of PA or Sedentary Time	Day 1 Thu.	Day 2 Fri.	Day 3 Sat.	Day 4 Sun.	Daily Average	Weekday Average	Weekend Day Average
Number of children with at least one valid day of data	42	40	43	28	38	41	36
Average daily wear minutes (*SD*)	754.2 (*76.6)*	765.8 (*101.3*)	721.1 (*90.6*)	738.4 (*99.8*)	722.1 (*71.5*)	748.2 (*81.1*)	716.6 (*83.4*)
Average daily non-wear minutes (*SD*)	685.0 (*77.1*)	673.2 (*100.9*)	717.9 (*91.2*)	702.2 (*98.9*)	748.2 (*153.2*)	758.5 (*89.0*)	761.3 (*95.1*)
Sedentary minutes (<0.01 kcal/kg/min; *SD*)	527.8 (*101.8*)	539.4 (*102.2*)	512.1 (*81.0*)	488.2 (*77.0*)	508.0 (*89.3*)	533.5 (*102.0*)	497.7 (*79.0*)
Moderate minutes (≥0.04 to <0.10 kcal/kg/min; *SD*)	53.0 (*34.8*)	48.0 (*34.3*)	41.2 (*32.3*)	62.2 (*38.1*)	46.6 (*31.3*)	49.1 (*34.6*)	47.0 (*35.3*)
Vigorous minutes (0.10 or more kcal/kg/min; *SD*)	6.1 (*8.3*)	2.2 (*4.3*)	4.0 (*10.1*)	3.2 (*4.3*)	3.4 (*6.1*)	3.8 (*6.3*)	3.8 (*7.2*)
Moderate-to-vigorous PA minutes (≥0.04 or more kcal/kg/min; *SD*)	58.9 (*41.1*)	50.0 (*35.8*)	45.3 (*41.2*)	64.0 (*38.7*)	50.0 (*34.2*)	52.9 (*38.5*)	50.6 (*40.0*)

## 4. Discussion

The purpose of the current present feasibility study was to examine the physical activity levels and sedentary time of a sample of Latino children between the ages of 10 and 14 years in London, Ontario, Canada. With regard to children’s physical activity levels, there are two sets of results that warrant discussion. Firstly, for all valid days of accelerometer wear time combined, the Latino children in our study spent an average of 54.4 min per day engaged in MVPA. While conclusions regarding Latino children’s habitual physical activity levels cannot be drawn on the basis of the poor adherence to the accelerometer protocol and the subsequent analyses, it is noteworthy that the abovementioned daily physical activity average approached the 60 min of MVPA per day recommended in the Canadian Physical Activity Guidelines [5].

The second set of physical activity results that is pertinent relates to sex differences. Results showed that boys were significantly more active than girls (albeit the significance for the objectively measured physical activity was considered “borderline” at *p* = 0.054). These findings are consistent with previous research with multi-ethnic Canadian [6], American [12], and Latino [11,29] children.

With regard to sedentary pursuits, two main sets of results should be highlighted. First, data findings revealed that Latino children spent close to nine hours per day being sedentary. This value was similar to the 7.7 h of daily sedentary time reported for Latino children in the United States [11], and consistent with the high levels of sedentary behaviors reported for Columbian children and youth in the 2014 Colombian Physical Activity Report Card [29]. Interestingly, there was no significant difference in the amount of time children spent engaged in sedentary behaviours on weekdays) *vs.* weekend days. Additionally, girls were found to be more inactive than boys, although again the difference was not statistically significant.

Secondly, Latino children in our study spent almost four hours per day in front of screens. In other words, almost half (44.2%) of the children’s total sedentary time was spent engaged in screen time. Colley and colleagues [6] have suggested that while the accelerometer data collected as part of the 2007–2009 CHMS was useful, it did not allow for a description of the behaviors that constituted children’s sedentary time. Thus, in the absence of contextual information about children’s sedentary behaviors, Colley *et al.* postulated that much of this time was likely devoted to screen-related behaviors [6]. Again, while we are unable to draw conclusions about Latino children’s habitual screen-related or other sedentary behaviors, the current findings from the CSBQ provide preliminary support for Colley and colleagues’ hypothesis.

The current findings regarding screen time are also consistent with studies of Latino children in the United States. For example, Feng and colleagues [30] examined a sample of Latino children aged 5 to 9 years and reported a mean total daily screen time of 3.4 h, and Eaton and colleagues [31] reported four hours of daily screen time among a sample of Latino youth in grades 9 through 12. It is also noteworthy that Fakhouri and colleagues [12] reported that Latino children (ages 6–11) were *more* likely to achieve screen time guidelines (*i.e.*, engage in less than two hours per day) than non-Hispanic children.

Finally, in addition to examining the physical activity levels and sedentary time of a sample of Latino children, another goal of the current study was to test the assessment procedures used with regard to their potential use in a larger-scale study examining the physical activity and sedentary behaviors of Latino children in Canada. As noted above, Latino children’s adherence to the accelerometer wear protocol was poor in the current study; of the 60 Latino children that were included in analyses, only one quarter of participants (*n* = 15) wore the device as instructed for the full four days. Given that researchers have suggested that accelerometer ‘nonwear’ is one of the many challenges associated with the use of such devices in community-based research (e.g., Sharpe and colleagues [32]), several strategies were used to promote adherence in the current study (*i.e.*, accelerometers were dropped off and picked up from participant’s homes, detailed instructions on how to use the accelerometer were provided to parents and participants by a trained research assistant, and participants completed daily wear logs). Clearly, additional strategies to improve accelerometer adherence rates including the potential use of reminder calls, a scripted and interactive accelerometer orientation, electronic and interactive wear-logs, and/or discussions of potential accelerometer-related challenges/issues [33] should be explored and utilized in future work with this young population. Interestingly, there has also been evidence of a “wear effect”, a temporary change in participant behaviors as a result of being observed [32]. Specifically, enhanced physical activity levels may be witnessed initially upon wearing an activity monitor, after which the levels return to more accurate values over time [32].

## 5. Conclusions

In summary, despite the fact that the Latino community represents one of the most sizeable non-European ethnic groups in Canada [16], little attention has been paid to the physical activity levels and sedentary behaviors of Latino children in this country. The current research represents an important step in addressing this gap by using measurement tools and methodology that, in combination, provide reliable quantitative and contextual information about physical activity *and* sedentary behaviors.

Additionally, while the current findings provide important preliminary evidence and a “snapshot” with regard to the self-reported and ‘daily’ physical activity levels and sedentary time of Latino children in London, Canada, the inclusion of children with only one valid day of accelerometer data prevent us from drawing conclusions with regard to habitual activity behaviors. This, in combination with our learnings regarding the accelerometer wear protocol, provide further impetus for the future investigation of the physical activity and sedentary behaviors of a larger sample of Latino children over a longer period of time, and with a comparison group.

This feasibility project provides the first account of objectively measured physical activity and sedentary behaviors among a sample of Latino children in Canada as well as insight into the challenge of measuring these behaviors. As mentioned above, the present study also offers novel insights into the *types* of sedentary pursuits that some Latino children engage in. As such, it is recommended that in addition to physical activity, researchers continue to explore sedentary behaviors and sedentary reduction strategies in this population.
